# Subthalamic nucleus phase–amplitude coupling correlates with motor impairment in Parkinson’s disease

**DOI:** 10.1016/j.clinph.2016.01.015

**Published:** 2016-04

**Authors:** Bernadette C.M. van Wijk, Martijn Beudel, Ashwani Jha, Ashwini Oswal, Tom Foltynie, Marwan I. Hariz, Patricia Limousin, Ludvic Zrinzo, Tipu Z. Aziz, Alexander L. Green, Peter Brown, Vladimir Litvak

**Affiliations:** a Wellcome Trust Centre for Neuroimaging, UCL Institute of Neurology, 12 Queen Square, WC1N 3BG London, United Kingdom; b Sobell Department of Motor Neuroscience and Movement Disorders, UCL Institute of Neurology, 33 Queen Square, WC1N 3BG London, United Kingdom; c Nuffield Department of Clinical Neuroscience, University of Oxford, John Radcliffe Hospital, OX3 9DU Oxford, United Kingdom; d University Medical Center Groningen, University of Groningen, Hanzeplein 1, 9713 GZ Groningen, The Netherlands; e Unit of Functional Neurosurgery, UCL Institute of Neurology, Queen Square, WC1N 3BG London, United Kingdom; f Department of Surgical Sciences, University of Oxford, John Radcliffe Hospital, OX3 9DU Oxford, United Kingdom; g Medical Research Council Brain Network Dynamics Unit at the University of Oxford, OX3 9DU Oxford, United Kingdom

**Keywords:** DBS, deep brain stimulation, HFO, high-frequency oscillations, LFP, local field potential, MEG, magnetoencephalography, PAC, phase–amplitude coupling, STN, subthalamic nucleus, UPDRS, Unified Parkinson’s Disease Rating Scale, Parkinson’s disease, Subthalamic nucleus, Cross-frequency coupling, Beta oscillations, Motor system, Local field potentials

## Abstract

**Objective:**

High-amplitude beta band oscillations within the subthalamic nucleus are frequently associated with Parkinson’s disease but it is unclear how they might lead to motor impairments. Here we investigate a likely pathological coupling between the phase of beta band oscillations and the amplitude of high-frequency oscillations around 300 Hz.

**Methods:**

We analysed an extensive data set comprising resting-state recordings obtained from deep brain stimulation electrodes in 33 patients before and/or after taking dopaminergic medication. We correlated mean values of spectral power and phase–amplitude coupling with severity of hemibody bradykinesia/rigidity. In addition, we used simultaneously recorded magnetoencephalography to look at functional interactions between the subthalamic nucleus and ipsilateral motor cortex.

**Results:**

Beta band power and phase–amplitude coupling within the subthalamic nucleus correlated positively with severity of motor impairment. This effect was more pronounced within the low-beta range, whilst coherence between subthalamic nucleus and motor cortex was dominant in the high-beta range.

**Conclusions:**

We speculate that the beta band might impede pro-kinetic high-frequency activity patterns when phase–amplitude coupling is prominent. Furthermore, results provide evidence for a functional subdivision of the beta band into low and high frequencies.

**Significance:**

Our findings contribute to the interpretation of oscillatory activity within the cortico-basal ganglia circuit.

## Highlights

• We obtained invasive subthalamic nucleus recordings in 33 Parkinson’s disease patients.
• Phase–amplitude coupling between beta band and high-frequency oscillations correlates with severity of motor impairments.
• Parkinsonian pathophysiology is more closely linked with low-beta band frequencies.

## 1 Introduction

Bradykinesia in Parkinson’s disease is typically associated with high-amplitude beta band (13–30 Hz) oscillations within the cortico-basal ganglia circuit (Gatev et al., 2006, Hammond et al., 2007, Oswal et al., 2013a). The subthalamic nucleus (STN) in particular forms one of the primary targets for deep brain stimulation (DBS) treatment of the disease. High-frequency stimulation around 130 Hz has proven highly effective in reducing symptoms of bradykinesia, rigidity and tremor, and in enhancing the quality of daily life (Krack et al., 2003, Rodriguez-Oroz et al., 2005, Deuschl et al., 2006, Weaver et al., 2009). Clinical improvement obtained with either DBS or the administration of dopaminergic medication seems closely related to its ability to reduce excessive beta band oscillations observed in the STN (Kühn et al., 2006, Kühn et al., 2008, Kühn et al., 2009, Weinberger et al., 2006, Ray et al., 2008, Eusebio et al., 2011). It remains, however, unclear how increased levels of beta band synchronisation mechanistically lead to motor impairments.

The beta band is not the only frequency band associated with Parkinson’s disease. Modulations induced by dopaminergic medication have also been observed for the theta–alpha range (4–12 Hz) (Alonso-Frech et al., 2006, Stoffers et al., 2008, Oswal et al., 2013a) and at gamma band frequencies (∼60–90 Hz) (Williams et al., 2002, Cassidy et al., 2002, Alegre et al., 2005, Kühn et al., 2006, Androulidakis et al., 2007, Lalo et al., 2008, Litvak et al., 2012). Moreover, there are indications for altered cross-frequency amplitude correlations between the theta and beta/gamma band during movement (Herz et al., 2014a, Herz et al., 2014b) and between the beta and gamma band in spontaneous activity (Fogelson et al., 2005). This implies that an integral perspective on activities throughout the entire frequency spectrum may be necessary to fully understand parkinsonian pathophysiology.

Recently, de Hemptinne et al. (2013) revealed a pathological coupling between the instantaneous phase of beta band oscillations and the amplitude of broad band gamma activity (50–200 Hz) in the primary motor cortex. This phase–amplitude coupling (PAC) was characteristic for Parkinson’s disease as it was absent for dystonia and epilepsy patients, and abolished during DBS (de Hemptinne et al., 2015). They observed a similar coupling between beta band oscillations recorded from the STN and broadband gamma activity in the primary motor cortex. The potential importance of PAC was further underscored by Shimamoto et al. (2013) who demonstrated that the magnitude of locking between spike activity in the STN and beta band oscillations in the primary motor cortex correlated with bradykinesia scores.

Local field potential (LFP) recordings obtained from DBS electrodes in the STN often contain yet another distinct spectral peak, falling in the range between 200 and 400 Hz (Foffani et al., 2003, López-Azcárate et al., 2010, Özkurt et al., 2011, Wang et al., 2014). We will refer to these as high-frequency oscillations (HFO). Its amplitude displays a clear movement-related increase that is more pronounced after levodopa administration (Foffani et al., 2003, López-Azcárate et al., 2010, Litvak et al., 2012). A small number of studies have shown that, during rest, fluctuations in the amplitude envelope of these HFO may be coupled to the phase of beta band oscillations extracted from the same LFP signal (López-Azcárate et al., 2010, Özkurt et al., 2011). Coupling strength was found to be higher before compared to after levodopa administration. Despite first observations by López-Azcárate et al. (2010) of a significant correlation between the strength of PAC and off medication Unified Parkinson’s Disease Rating Scale (UPDRS) scores, it remains to be established to what extent PAC contributes to the symptoms of Parkinson’s disease.

To investigate the relation between beta/HFO PAC and severity of motor impairment in more detail, we analysed a large cohort of patients for whom we acquired LFP recordings from the STN in combination with simultaneous magnetoencephalography (MEG). Using hemibody bradykinesia/rigidity UPDRS scores, we were able to demonstrate a significant correlation for both beta band power and PAC in the STN. In addition, we provide further evidence for a functional subdivision between low- and high-beta frequencies by comparing patterns of phase–amplitude coupling within the STN and beta band coherence between STN and ipsilateral motor cortex.

## 2 Material and methods

### 2.1 Patients and surgery

All patients underwent STN electrode implantation for DBS treatment of Parkinson’s disease at the National Hospital of Neurology and Neurosurgery (University College London) or the John Radcliffe Hospital (University of Oxford) between 2007 and 2014. Ethical approval was obtained from the local ethics committees at University College London and University of Oxford, and 33 subjects gave written informed consent to the study, and were recorded. All were diagnosed with Parkinson’s disease according to the Queen Square Brain Bank Criteria (Gibb and Lees, 1988). Motor impairments of each patient were evaluated on average approximately 5 months prior to surgery using part III of the UPDRS after omitting all dopaminergic medication overnight, and following administration of at least 200 mg of levodopa. For detailed patient characteristics see Supplementary Table S1. Data from a subset of these patients have been described in earlier publications (Litvak et al., 2011a, Litvak et al., 2012, Oswal et al., 2013a).

**Supplementary Fig. S1:**
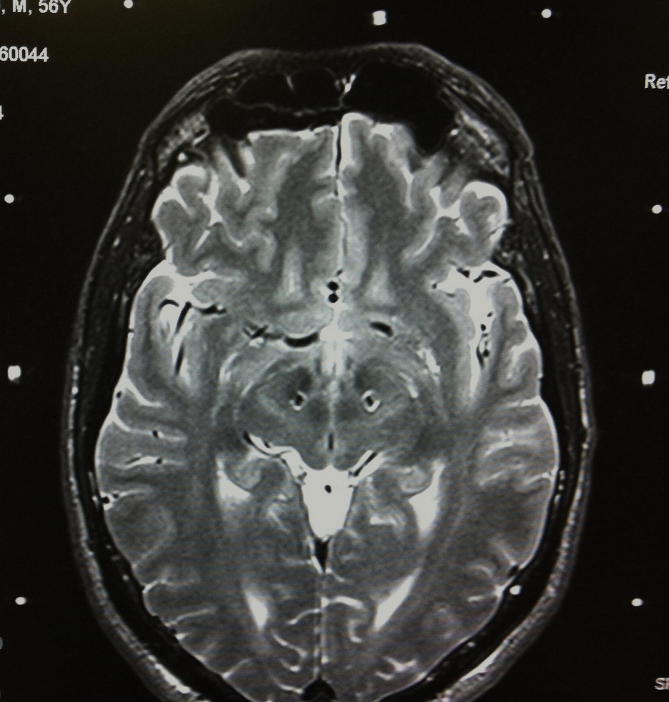
Immediate post-implant stereotactic axial T2-weighted MR scan at the level of the lowest contact of the DBS electrodes lying in the posterior part of the ventral STN. Electrodes were connected to an accessory kit, typically both connectors being tunnelled to the left temporoparietal area and externalized through the frontal region. Electrode locations were confirmed using postoperative stereotactic imaging

The surgical procedure involved the bilateral implantation of deep brain stimulation electrodes each with four contacts (model 3389, Medtronic, Minneapolis, MN, USA). In one patient implantation was carried out unilaterally. In London, the intended target for the lowermost contact (contact zero) was determined on the stereotactic axial T2-weighted MRI scan lying at the level of the uppermost part of the mammillothalamic tract, which corresponds to the ventral part of the STN as visualised on the MRI (Hariz et al., 2003, Foltynie et al., 2011). An immediate post-implantation stereotactic T2-weighted MRI was performed to verify electrode location (see Supplementary Fig. S1). Electrodes were connected to an accessory kit, typically both connectors being tunnelled to the left temporoparietal area and externalised through the frontal region. For more details see Litvak et al. (2011a). In Oxford, the targeting was the same but was performed on a pre-operative volume-based MRI using T2-weighted and FLAIR sequences to target the dorsolateral STN at the level of the red nucleus with advancement of the electrode 3–5 mm caudally. The MRI was fused to an intraoperative stereotactic CT head scan to obtain the coordinates and a post-operative check CT scan was also fused to the pre-operative MRI.

### 2.2 Data recordings and preprocessing

Simultaneous recordings were obtained from the deep brain stimulation electrodes and 275-channel MEG (CTF/VSM MedTech, Vancouver, Canada) for all patients between two and six days postoperatively. At this time, continuous DBS for treatment purposes had not yet started. Data were online low-pass filtered at 600 Hz, sampled at 2400 Hz, and stored to disc. For a subset of cases (29–33) STN–LFPs were recorded using an EEG system separate from the MEG. MEG and LFPs were later temporally realigned and fused using a white noise reference signal recorded on both systems. A more comprehensive description of this methodology is provided in Oswal et al. (2015). Although the experiment also involved simple movement tasks for some of the patients, we here only analyse recordings obtained during periods of rest lasting about 3 min. Subjects were instructed not to move during the recordings and to focus their attention on a fixation cross without performing any explicit task. Separate experimental sessions were performed, for 13 out of 19 subjects on subsequent days, whilst the patient was either “ON” or “OFF” their normal dose of dopaminergic medication (after overnight withdrawal for the OFF state). The order of these sessions was counterbalanced across subjects. For a minority of subjects we were only able to obtain ON state recordings (5 cases) or OFF state recordings (9 cases). In 17 out of 53 recording sessions subjects performed the 3-min resting block twice, for the other sessions we had one block available.

We first focus our account on the analyses conducted for the LFP recordings obtained from the DBS electrodes, those for the MEG data are described later. DBS was switched off during the experiments, allowing for the acquisition of LFPs from all four contact points of the STN electrodes. These were off-line converted to a bi-polar montage between adjacent contacts, resulting in 3 channels per site. This was done to limit volume conduction of signals form distal sources. 50 Hz line noise and its harmonics up to 600 Hz were filtered out using 4th order Butterworth bandstop filters with cutoff frequencies ±0.5 Hz around the centre frequencies. We subsequently divided the continuous time series into epochs of 3.41 s and discarded epochs where the peak-to-peak LFP amplitude exceeded 100 μV. On average, 60 epochs (range 13–106) remained per STN site for further analyses. We included all available data in our analyses. This amounted to a dataset comprising recordings from 53 hemispheres with patients OFF medication and 48 hemispheres with patients ON medication.

### 2.3 Measures of spectral power and phase–amplitude coupling

We computed power spectral densities up to 400 Hz to see if we could replicate earlier findings of beta band reduction with dopaminergic medication and whether we could observe HFO in the recordings. Furthermore, we used the power spectra to determine if PAC could explain any variance in clinical scores independently from spectral power alone. We used Matlab’s (R2014a, The Mathworks Inc., Natick, USA) *pwelch*.*m* function to compute power spectral densities for each epoch (using default values of 8 Hamming windows with 50% overlap), and then averaged spectra across epochs. Values for frequencies ±3 Hz around 50 Hz harmonics were removed from the spectra in order to avoid the influence of notch filters on summary measures of spectral power. Power values for frequencies below 50 Hz were normalised by dividing by the power summed over all frequencies in the entire spectrum for each subject, condition, and bipolar STN channel individually. Values within the 150–400 Hz interval were divided by the mean power between 400 and 500 Hz to eliminate inter-subject variability in offset values in this frequency range.

For the computation of PAC we followed the parametric approach described in Penny et al. (2008) and van Wijk et al. (2015). Specifically, we first obtained low- and high-frequency components of the LFP signals by bandpass-filtering around centre frequencies between 5 and 35 Hz with 0.5 Hz steps (filter bandwidth ±1 Hz), and between 150 and 400 Hz in steps of 2 Hz (filter bandwidth ±35 Hz). Subsequently we extracted the instantaneous phase for each low-frequency component via $\theta_{x}=\text{mod}\left(\text{angle}\left(\text{hilbert}\left(x\right)\right)\text{,}2\pi \right)$, and amplitude of each high-frequency component via $a_{y}=\mathrm{abs}\left(\text{hilbert}\left(y\right)\right)$. To avoid filter ringing we removed the first and last 167 ms of each epoch. PAC was then estimated for each low-/high-frequency pair of frequency combinations using a general linear model of the form:

$$a_{y}=\beta_{1}\sin(\theta_{x})+\beta_{2}\cos(\theta_{x})\text{,}$$

where we took $r=\sqrt{\beta_{1}^{2}+\beta_{2}^{2}}$ as the strength of PAC. We performed this analysis in two ways for each individual STN channel: (1) with time series concatenated across all epochs to assess the overall PAC; (2) for each epoch individually. The latter results in a set of β-coefficients, which we used to test for significance of the overall PAC via an *F*-test. The actual PAC values reported in the paper were estimated after concatenation of time series across epochs. More details of this method can be found in van Wijk et al. (2015). The corresponding Matlab code is implemented within the open-source SPM toolbox (http://www.fil.ion.ucl.ac.uk/spm/, Litvak et al., 2011b).

We were interested in the association between the severity of motor impairment and activity within the beta band (13–30 Hz) and HFO range (150–400 Hz). As our results suggested differential modulations for low and high frequencies within the beta band, we decided to divide it into a range of low-beta and a range of high-beta frequencies and to consider them separately in subsequent analyses. The cut-off frequency between low- and high-beta frequencies was determined based on the histogram of all beta band peak and sub-peak frequencies of the power spectrum, which showed a clear bimodal pattern. A mixture of two normal distributions was fitted and the lowest point between the centre frequencies served as the cut-off frequency. As a result, the low-beta range comprised frequencies between 13 and 20.36 Hz, and the high-beta range between 20.36 and 30 Hz. Mean power and PAC values were obtained by averaging across all frequencies within the specified ranges for low/high-beta and/or HFO.

Due to the small spatial volume of the STN, we expect some variability in the precise location of the contacts within the STN with some contacts possibly even being outside the STN borders. Nevertheless we decided not to bias results by pre-selecting the channels with the strongest spectral power or PAC as we observed that these could occur at different channels. We therefore averaged all measures across all three bipolar channels for each electrode, resulting for each subject in mean values for both left and right STN, and ON and OFF medication conditions, when data were available. For visualisation of the grand-average power spectral densities, we employed robust averaging to eliminate the influence of outliers (Wager et al., 2005).

Finally, we obtained the mean phase angle for the phase and amplitude frequency combination at which PAC was highest within the spectrum. We only considered the channel with the highest peak PAC per STN in order to avoid the introduction of additional phase lags for channels located further away from the PAC generation site. We tested whether the distribution of phase angles across subjects significantly different from a uniform distribution using a Rayleigh test.

### 2.4 Correlation with clinical scores

Our measures of spectral power and PAC hence included: mean low-beta power, mean high-beta power, mean low-beta/HFO PAC, mean high-beta/HFO PAC, and mean HFO power. We were interested in their predictive value in explaining the severity of motor impairments manifest contralateral to the STN sites. These impairments were quantified via UPDRS scores of hemibody bradykinesia and rigidity for both ON and OFF medication states. Left/right hemibody scores were derived as the sum of the following items: 3.3 rigidity upper extremity, 3.3 rigidity lower extremity, 3.4 finger tapping, 3.5 hand movements, 3.6 pronation–supination movements of hands, 3.7 toe tapping, and 3.8 leg agility. We considered left and right STN sites from individual patients as independent samples, as well as values obtained during the ON and OFF medication state for each patient. These were combined into a single data vector comprising 101 samples. This resulted in a large range of UPDRS scores, which may be more suitable for revealing significant correlations in the presence of large inter-subject variability in power and PAC values.

Pairwise correlations were computed between all variables. In addition, given the large covariation between spectral power and PAC, we also performed a multiple regression analysis with contralateral bradykinesia/rigidity UPDRS scores as independent variable and beta band power, HFO power, and PAC as predictors. Analyses were repeated for the difference between OFF and ON states (OFF–ON), meaning that we directly correlated the clinical improvement obtained after levodopa administration with the observed change in spectral power and PAC measures. For this, we could only include patients with both OFF and ON recordings available, leaving 38 STN sites from 19 subjects.

### 2.5 Coupling between subthalamic nucleus and motor cortex

In a subset of 13 patients we have previously reported beta band coherence between STN and ipsilateral motor cortical areas (Litvak et al., 2011a). Here we investigated whether the phase of beta band oscillations in the motor cortex was coupled to the amplitude of HFO in the STN in these patients. We included cases who showed significant coherence with ipsilateral motor areas (cases 2–7, 9–13, 15 and 16 in Supplementary Table S1). For each subject and STN channel, we selected the MNI location of peak coherence and extracted the source time series for this location using a beamforming approach. This has been shown to successfully eliminate artefacts originating from the percutaneous DBS electrode wires (Litvak et al., 2010). A single-shell head model was used to compute the lead fields for the forward model (Nolte, 2003). These were constructed based on the subject’s preoperative MRI image that was normalised to a canonical template MRI scan. Raw time series were band-pass filtered between 5 and 35 Hz prior to computation of the covariance matrix. As explained in (Litvak et al., 2011a) we used the imaginary part of the cross-spectral density to obtain a single time series for a resultant dipole orientation per source. We computed the full PAC spectrum between beta and HFO frequencies for each pair of STN channel and corresponding cortical location using the same settings for PAC within the STN, as described above.

In order to directly compare beta–HFO PAC with beta band coherence, we recomputed the coherence spectra using Matlab’s *mscohere*.*m* function with setting analogous to the computation of spectral power (see above). Resulting spectra were grand averaged for OFF and ON medication conditions separately.

## 3 Results

### 3.1 Grand-average phase–amplitude coupling and spectral power

**Fig. 1:**
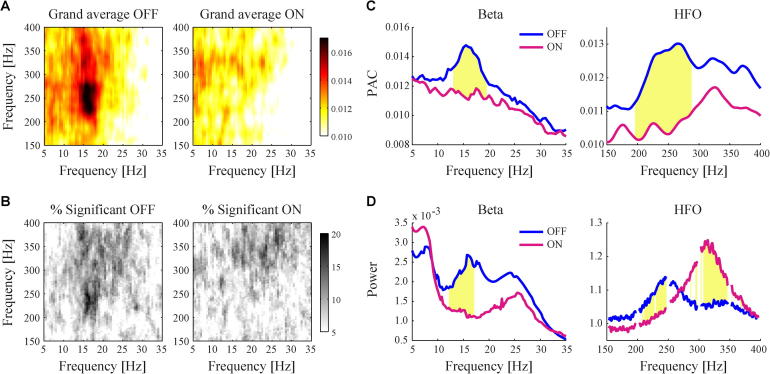
Grand-average PAC and power spectral densities separated by OFF and ON dopaminergic states. (A) Higher PAC values were observed in the OFF state, which were centred around frequencies in the lower beta and HFO range. (B) Percentage of cases with significant PAC (*p* < .05) for all beta and HFO frequency combinations in the spectrum. (C) PAC averaged across all HFO frequencies (left) or across all beta frequencies (right) was significantly higher for lower beta band frequencies in the OFF (blue) compared to ON (purple) medication state, and showed a shift in peak frequency within the HFO range. Yellow patches indicate for which 1 Hz frequency bins significant differences were detected between OFF and ON as determined with independent samples t-tests (*p* < .05). (D) The grand-average power spectral densities expressed similar modulations as PAC. (For interpretation of the references to colour in this figure legend, the reader is referred to the web version of this article.)

The majority of subjects showed clear significant beta–HFO PAC in either left or right STN, OFF or ON medication state. PAC averaged across all sides and subjects was higher in the OFF state compared to the ON state, due to the appearance of a high peak for phase frequencies in the lower beta range (Fig. 1A). The frequency of the phase modulated HFO was also lower in the OFF than ON medication state. The same pattern can be observed when looking at the percentage of cases showing significant PAC (Fig. 1B), as determined by our parametric estimation of PAC (see van Wijk et al., 2015). These medication-induced modulations suggest that both the strength of PAC and the combination of phase and amplitude frequencies at which it occurs could be important markers for the severity of motor impairment.

Fig. 1(C) shows the profile of PAC across beta band and HFO frequencies separately, i.e. when averaged across all amplitude frequencies or phase frequencies. There is a striking correspondence between these curves and those for the power spectral densities in the corresponding frequency ranges (Fig. 1D). In particular, there was a spectral power peak in the low beta range that corresponded to the peak frequency of phase modulation of HFO in the OFF medication state. This low beta activity in power and PAC was significantly suppressed by medication. By contrast, the grand-average spectral density demonstrated a second peak in the upper beta band, which was not associated with phase modulation of HFO and was less responsive to medication. At higher frequencies the grand-average spectral densities demonstrated a peak centred at 250 Hz OFF medication, which shifted to 320 Hz ON medication. This was roughly paralleled by changes in the frequency of phase modulated HFO. Similar shifts in peak frequency have been observed by (López-Azcárate et al., 2010, Özkurt et al., 2011).

**Fig. 2:**
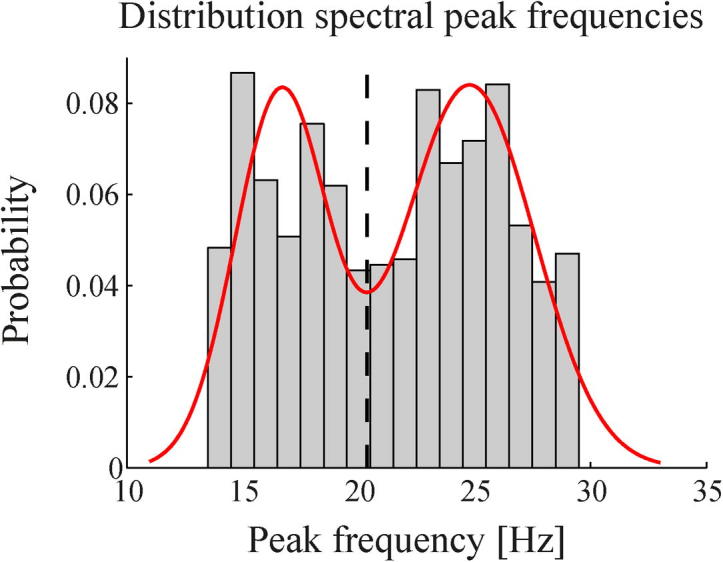
Histogram of peak frequencies within the spectral beta band. All spectral peaks and sub-peaks within the beta band were included for all subjects, conditions, and STN channels. This revealed a clear bimodal pattern. Overlaid in red is the optimal fit of a mixture of two normal distributions. The first distribution had its mean at 16.6 Hz and a standard deviation of 1.99 Hz, the second distribution at 24.79 Hz and a standard deviation of 2.81 Hz. The lowest point between distributions (20.36 Hz) was taken as the cut-off frequency between low- and high-beta ranges. (For interpretation of the references to colour in this figure legend, the reader is referred to the web version of this article.)

In order to substantiate the differential effects between low and high beta band frequencies, we considered them separately in further analyses. The histogram of all beta band peak and sub-peak frequencies in the individual power spectra showed a clear bimodal pattern (Fig. 2). The optimal fit of a mixture of two normal distributions was obtained for centre frequencies at 16.65 and 24.79 Hz, with standard deviations of 1.99 and 2.81 Hz, respectively. The cut-off frequency between low- and high-beta ranges was found to be 20.36 Hz. By comparison, the difference in Akaike information criterion value with a fit of a single normal distribution was 169, significantly favouring the bimodal over the unimodal distribution.

### 3.2 Relation between PAC and spectral power

**Fig. 3:**
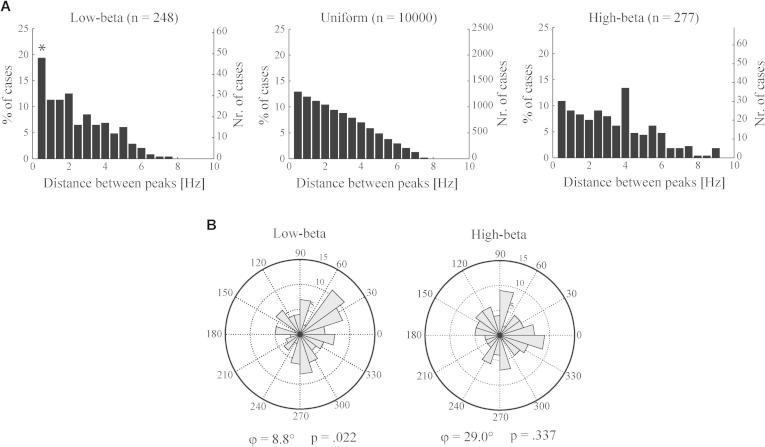
Distribution of peak frequencies and phase angles within the low- and high-beta range. (A) Histograms show the distance between peak frequencies for PAC and corresponding power spectral densities within individual channels. Bins represent 0.5 Hz intervals. The observed number of cases for each bin was compared against 1000 surrogate histograms obtained by randomly reshuffling the PAC peak frequencies within the low- or high-beta ranges. Spectral peak frequencies were left at their observed values. Bins exceeding the .05 or .95 percentiles are indicated with an asterisk. For the low-beta range (left), a significantly large proportion of samples were found to have PAC and spectral peaks within 0.5 Hz proximity. For visualisation, a histogram is plotted for the pair-wise distance between a large number of uniformly distributed frequencies within the low-beta range (middle). The high-beta range did not show an exceedingly large proportion of samples with peaks in close proximity (right). (B) Distribution of phase angles computed for the beta–HFO frequency combinations for which PAC was maximal. Only one channel per STN site was taken into account, resulting in 101 samples considered. A significant non-uniform distribution was found only for the low-beta range: mean angle = 8.8° (*p* = .022).

PAC and beta band power were modulated by dopaminergic medication in a very similar way. Indeed, a strong correlation between their mean values was found for both the low- and high-beta range (Table 1). We next considered whether this relation also holds regarding the exact frequencies at which their peak values occur. Most PAC involved modulation of HFOs by the phase of the low beta band activity, and within this range the phase frequency corresponding to the highest PAC value tended to coincide with or was close to the spectral peak frequency (Fig. 3A). Analogous histograms for the high-beta range showed less consistency between the frequency of highest PAC and spectral peak frequencies. Similarly, the distribution of mean phase angles only significantly differed from a uniform distribution for the low-beta range (mean = 8.8°, *p* = .022) but not the high-beta range (Fig. 3B). These findings indicate a larger variability in PAC for the high-beta range. Group-level PAC mainly occurred within the low-beta range, and predominantly for frequencies close to the peak in the power spectrum.

Regarding HFO, cases with significant PAC were only found if there was also a clear HFO peak observable in the power spectrum. The amplitude frequency of maximum PAC was always relatively close to the peak frequency of HFO power. No correlations were found between HFO power and PAC or beta power (Table 1).

### 3.3 Correlations with clinical scores

**Fig. 4:**
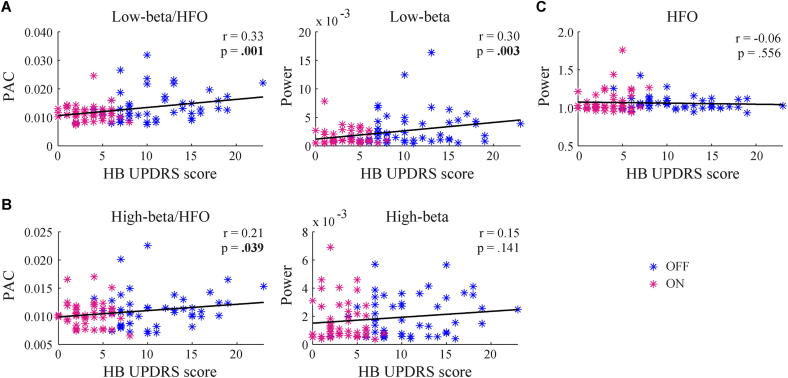
Regression analyses between hemibody bradykinesia/rigidity UPDRS scores and mean beta/HFO power and PAC separately. Samples taken from the OFF medication state are depicted in blue, samples from the ON state in purple. Note that OFF and ON states were combined in the computation of the regression coefficients. Correlation coefficients and *p*-values are indicated for each plot with significant relations in bold. (A) Low-beta range; (B) High-beta range; (C) HFO. (For interpretation of the references to colour in this figure legend, the reader is referred to the web version of this article.)

**Fig. 5:**
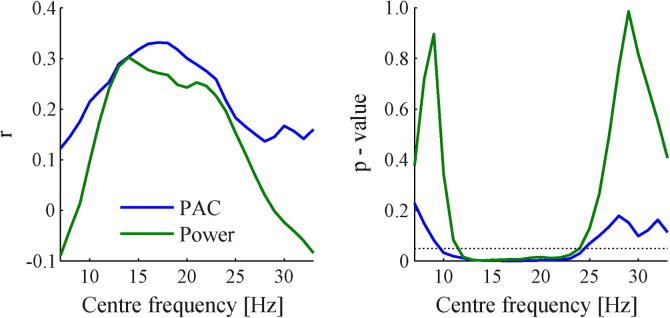
Correlations between UDPRS scores and PAC/power across different frequency ranges. Pair-wise correlations were performed between hemibody bradykinesia/rigidity UPDRS scores and either PAC or spectral power using a moving frequency window with 4 Hz width and centred on frequencies from 7 to 33 Hz with 1 Hz steps. Correlation coefficients *r* were highest and *p*-values lowest for centre-frequencies in the lower-beta range. Correlations ceased to be significant towards the theta/alpha range, as well as the higher beta band.

We tested whether mean PAC and spectral power values could explain some of the variability in contralateral hemibody bradykinesia/rigidity UPDRS scores. OFF and ON medication states were combined in this analysis to increase the range of UPDRS scores. Separate regression analyses were performed for the low- and high-beta ranges. Results are visualised in Fig. 4 and all correlation coefficients and *p*-values are reported in Table 1. We found a significant positive correlation for PAC and spectral power in the low-beta range, indicating higher PAC and power values with more severe bradykinesia and rigidity symptoms. For the high-beta range, the correlation with spectral power no longer reached significance and the correlation with PAC did not survive a Bonferroni correction for the number of pair-wise correlations performed. Additional analyses revealed that correlation coefficients were highest and *p*-values lowest for frequency windows centred in the low-beta range (Fig. 5). No significant correlations were found for HFO spectral power.

Given the strong correlation between mean PAC value and beta power, we performed a single multiple regression analysis with UPDRS scores as dependent variable and beta/HFO power and PAC as predictors. Although this analysis failed to reveal a significant independent contribution for any of the variables (Table 2), the partial correlation for PAC was higher compared to beta power and showed a trend towards significance (*p* = .066).

Correlations between improvement in clinical scores and changes in mean power and PAC values between OFF and ON medication states were also estimated for the two beta ranges. This reflects whether individual clinical improvement was associated with within-patient changes in power/PAC. In general, similar trends can be observed as for the absolute values but effects were found to be weaker (Table 3, Table 4). Although correlation coefficients for PAC and spectral power in the low-beta range were almost as high as for the absolute values, their *p*-values no longer indicated a significant relation. This is possibly due to a loss of statistical power, given the large reduction in the number of samples.

### 3.4 Coupling between STN and motor cortex

**Fig. 6:**
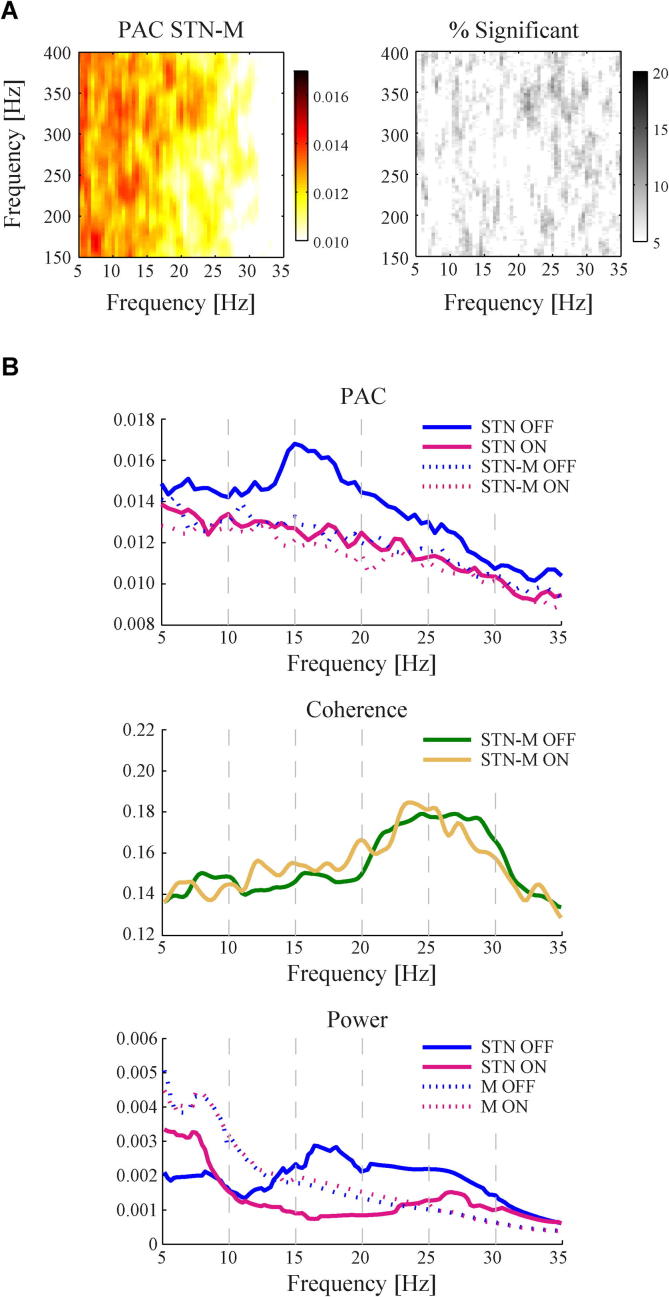
Distinct beta frequency ranges for STN activity and STN-motor cortex interactions. (A) Grand-average PAC between HFO in the STN and beta band activity in the motor cortex. Spectra are averaged across OFF and ON medication states and left/right STN channels in 11 subjects, comprising a total of 128 recordings. Unlike beta–HFO PAC in the STN, PAC between STN and motor cortex (STN-M) was found to be weaker and large clusters of significant beta/HFO frequency combinations were rarely detected. (B) Differential effects in PAC and coherence within the low- and high-beta range. Beta–HFO PAC within STN was mainly strong for low beta band frequencies, whereas beta band coherence between STN and motor cortex centred around high beta frequencies. This might explain why no distinct PAC was found between the phase of beta band cortical activity and HFO amplitude in the STN. The strongest modulations in spectral power with dopaminergic treatment were also found in the lower beta frequency range within the STN.

Finally, we investigated whether the amplitude of HFO in the STN was also locked to the phase of beta oscillations in the motor cortex. The grand average across all recordings is shown in Fig. 6(A). In general, PAC between STN and cortical motor areas was found to be weaker compared to PAC within STN and distinct patterns within the spectrum were only rarely observed. For example, we only found 1 case in which a cluster of >400 adjacent significant points of beta/HFO frequency combinations could be detected, compared to 13 cases for PAC within STN for this subgroup of subjects. A possible explanation for the lack of clear STN-cortex PAC emerges when we directly compare the within-STN PAC and the STN-cortex beta coherence (Fig. 6B). This reveals that PAC within the STN mainly occurs for lower beta band frequencies (∼17 Hz), whereas STN-cortex coherence is dominated by higher beta band frequencies (∼25 Hz). No significant correlations were found between the UPDRS scores and average STN-cortex PAC in the low-beta or high-beta band range, nor for cortical power or coherence.

## 4 Discussion

Using a relatively large cohort of patients, we have demonstrated that phase–amplitude coupling between beta band activity and HFO in the subthalamic nucleus of Parkinson’s disease patients correlates with severity of bradykinesia/rigidity. Higher PAC values were associated with stronger motor impairment. Despite the strong correlation between PAC and beta band spectral power, results suggested that PAC might have slightly superior predictive value in explaining the variability in measured UPDRS scores. We found little evidence for a coupling between HFO in the STN and beta band activity in cortical motor areas. A dissociation was made between low- and high-beta frequency ranges as medication-induced effects on PAC and spectral power within the STN were found to be confined to the former, whereas coherence between the STN and cortical motor regions was predominant in the latter.

### 4.1 A pathological role for PAC

Phase–amplitude coupling has more commonly been ascribed a functional role for information processing within the brain (Jensen and Colgin, 2007). Our findings indicate that PAC may also serve as a pathological mechanism. One intriguing explanation for why exaggerated levels of beta–HFO PAC in the STN might lead to motor impairments is that the pro-kinetic HFO become locked by the beta band oscillation, which prevents them from initiating firing patterns underlying movement (López-Azcárate et al., 2010). Our partial correlations support this theory, demonstrating that UPDRS scores were more likely to be directly linked to PAC values than to beta power. Related to this, alpha–gamma coupling in the motor cortex has been found to decrease in anticipation of an upcoming cued movement (Yanagisawa et al., 2012). Hence the reduction of coupling with lower frequencies could free up resources for movement-related activity.

If a high level of beta oscillations means that neurons involved in HFO are more constrained in their firing patterns, one could speculate that the level of PAC is a direct consequence of the amplitude of beta oscillations. Indeed, we found a highly significant positive correlation between the two. A complicating confounding factor in this discussion is the influence of signal-to-noise ratio, which may affect the accuracy of beta phase estimates and, in consequence, PAC. However, beta peak frequencies for individual power and PAC spectra did not overlap exactly. Also, permuting the order of trials for which phase time series were extracted, as typically done for non-parametric tests, destroyed the distinct patterns of significant PAC (results not shown), suggesting that it is not the level of beta power as such that leads to higher PAC estimates but its interaction with HFO.

### 4.2 Neurophysiology of HFO

To date, surprisingly little is known about the neuronal origins of HFO. Although the high frequency range suggest a reflection of multiunit spiking activity, the fact that a clear and relatively narrow peak can be observed in the power spectrum implies an oscillatory component. In order to resolve this issue, Yang and colleagues (2014) simultaneously recorded single unit activity and local field potentials from a pair of micro- and macroelectrode contacts during DBS surgery of the STN. Notably, both neuronal spiking and HFO were locked to the phase of beta band oscillations but their occurrence was uncorrelated, suggesting that spiking activity does not directly contribute to beta/HFO as such. This has been further corroborated by Wang et al. (2014) who showed that the removal of spikes from the microelectrode time series marginally affected the spectral power of HFO.

Similar HFO between 200 and 300 Hz have been found in Parkinson’s disease patients with DBS electrodes implanted in the internal part of the globus pallidus (GPi) (Tsiokos et al., 2013). Like HFO in the STN (Foffani et al., 2003, López-Azcárate et al., 2010, Litvak et al., 2012, Tan et al., 2013), the amplitude of this activity increases during movement. Also, Connolly et al. (2015) showed that PAC between HFO and beta oscillations in the GP emerges when rhesus macaque monkeys are rendered parkinsonian with 1-methyl-4-phenyl-1,2,3,6-tetrahydropyridine (MPTP) injections. PAC gradually increased with higher MPTP dosages, which were accompanied by more severe parkinsonian states.

Single neurons within the STN may show bursting activity with intraburst firing rates roughly between 100 and 300 Hz (Wichmann and Soares, 2006, Gale et al., 2009, Sanders et al., 2013, Sharott et al., 2014). Some of the previously reported modulations of bursting activity seem to be in line with those for HFO. Like the spectral amplitude of HFO, the number of bursts increases with movement (Gale et al., 2009). Also, bursting occurs in a slightly more oscillatory fashion in the parkinsonian state (Wichmann and Soares, 2006). However, other findings are more difficult to reconcile. For example, it is known that bursting occurs more frequently in parkinsonian states compared to normal physiological conditions (Lobb, 2014), but we did not find a significant correlation between the spectral amplitude of HFO and UPDRS scores nor did we observe a difference in mean HFO power between ON and OFF medication states (independent *t*-test: *t*(97) = 0.702, *p* = .484). Also, the intraburst frequency is higher in the parkinsonian state (Sanders et al., 2013, Sharott et al., 2014), whereas we observed the opposite pattern (median HFO frequency was higher for ON versus OFF medication, independent *t*-test: *t*(97) = 3.151, *p* = .002). More work is needed to determine whether HFO as observed in multiunit recordings directly relates to bursting discharge patterns of single neurons.

### 4.3 Differential roles for low and high beta frequencies

In a previous study using a subset of the data presented here (Litvak et al., 2011a), we found that dopaminergic treatment mainly affected low-beta (∼15–20 Hz) spectral power in the STN. By contrast, coherence between STN and activity in (pre)motor cortex centred around higher beta band frequencies (∼23–28 Hz) and was not modulated by levodopa. Here we extend these findings with the observation that beta–HFO PAC within the STN was largely confined to the low-beta range. This further strengthens the notion that there might be a functional subdivision within the beta band, with beta activity at lower frequencies being more conspicuously related to the hypo-dopaminergic state of Parkinsonism (Fogelson et al., 2006, Litvak et al., 2011a, Little et al., 2013b). In contrast, communication between STN and motor cortex seems to be mediated by high-beta frequencies, and it is unresolved whether this higher frequency beta activity is a marker of parkinsonian pathophysiology (Oswal et al., 2013b), leaving the possibility that it may form part of normal physiological network activity. Although we did not find clear evidence for PAC with beta oscillations in the motor cortex, it could be that recordings with a higher signal-to-noise ratio, such as obtained with electrocorticography, will be able to reveal this. However, at present we are unaware of reports by others showing that there is such coupling.

### 4.4 Finding biomarkers of Parkinson’s disease

Typical observations from single unit recordings obtained from the STN or GP in human patients with Parkinson’s disease or parkinsonian rendered animals include an increased neuronal firing rate, an increase in bursting activity, and more synchronised spiking activity (Galvan and Wichmann, 2008). The latter can also readily be observed in multiunit recordings as exaggerated levels of beta band oscillations. A recently identified biomarker is the occurrence of beta–gamma phase–amplitude coupling within primary motor cortex (de Hemptinne et al., 2013). We have demonstrated that beta–HFO PAC within STN can be added to the list of indicators of parkinsonian motor severity, albeit one that is highly related to beta band power.

Correlation coefficients with motor impairment were relatively low (maximum value was found to be 0.33). The UPDRS scores assessed months before surgery are unlikely to have been completely representative of the clinical state at the time of recording due to the temporary post-operative improvement caused by the stun effect. Tykocki et al. (2013) examined the magnitude of this ‘microlesion effect’ in a large cohort of patients (*n* = 74), and found that total UPDRS III scores assessed within 48 h post-operatively decreased by 17.9% on average (standard deviation of 15.7%). The relatively long time period between the acquisition of the UPDRS scores and the actual recordings may have introduced additional variability in the relation with power and PAC measures. For these reasons, the actual relation between PAC and motor impairment might be stronger than we observed. On the other hand, low coefficients may indicate that constraining effects of the low-beta rhythm on HFO activity patterns might be only one of several mechanisms in which motor impairments could arise. It is also thinkable that PAC and/or power are epiphenomenal to a more direct neuronal cause of motor impairments. Our analyses merely point at associations for the magnitude of PAC and beta power but do not allow to pinpoint them as the direct cause.

The magnitude of PAC and spectral power detected in our recorded signals might have depended on the positioning of the electrodes with respect to the generating source. It is even possible that beta oscillations and HFO may originate in slightly different spatial locations (Wang et al., 2014). Variability across subjects in targeting the right compartment of the STN could have influenced both absolute and relative spectral values. PAC on the other hand, might be less sensitive to this issue as it is a normalised measure based on the variance of amplitude fluctuations present in the signal. Given that the difference in correlations coefficients for the relation with UPDRS scores were relatively small between PAC and beta power, the predominance of one over the other should be carefully interpreted.

The identification of biomarkers for Parkinson’s disease is crucial for understanding its pathophysiology, as well as for development of effective treatments. Current efforts to optimise DBS treatment use the amplitude of beta band oscillations as a signal feature to be controlled in an adaptive/closed-loop setting (Little et al., 2013a). Given that beta band power can be both more readily picked up (due to the higher amplitude of beta activity over HFO) with ultra-low power amplifiers and more readily computed from recorded time series, we believe that it is potentially more suitable to use in adaptive DBS applications than beta–HFO PAC. However, our findings make important contributions to understanding the neuronal mechanisms that might lead to movement impairments. The robust link between low-beta power and HFO suggests that beta band oscillations constrain the pro-kinetic HFO to stereotypic activity patterns. Reducing low-beta power might indirectly lead to an improvement of symptoms by facilitating the initiation of movement-related activity.

## Figures and Tables

**Table 1:** Pairwise correlations between all variables for absolute values.

Correlation coefficients *r* and corresponding *p*-values are reported. The upper triangle lists correlations amongst variables in the low-beta range (with samples for OFF and ON combined in a single data vector), correlations between variables in the high-beta range are listed in the lower triangle. Significant values are indicated in bold. Note that the correlation coefficient for mean PAC in the high-beta range is considerably lower compared to the low-beta range, and would not survive a Bonferroni correction for the number of correlations performed per range (*α* = .05/6 = .0083).

**Table 2:** Multiple regression results with hemibody bradykinesia/rigidity UPDRS scores as dependent variable.

	PAC	Beta Power	HFO Power
*Low-beta*			
*r*_partial_	0.19	0.11	−0.10
*p*-value	.066	.300	.346

*High-beta*			
*r*_partial_	0.18	0.09	−0.08
*p*-value	.077	.370	.443

No significant contributions were found.

**Table 3:** Pairwise correlations between all variables for changes between OFF and ON medication states.

Correlation coefficients *r* and corresponding *p*-values are reported. Significant values are indicated in bold. Correlations were performed on the change in variables between OFF and ON medication states. The upper triangle lists correlations within the low-beta range, correlations for the high-beta range are listed in the lower triangle.

**Table 4:** Multiple regression results for changes in variables between OFF and ON medication states.

	PAC	Beta Power	HFO Power
*Low-beta*			
*r*_partial_	0.20	0.11	−0.11
*p*-value	.259	.545	.525

*High-beta*			
*r*_partial_	0.21	0.02	−0.05
*p*-value	.227	.915	.770

No significant contributions were found.

## Appendix A Supplementary data

Supplementary Table S1 Patient characteristics.
