# Supplementary material for: Subthalamic nucleus phase–amplitude coupling correlates with motor impairment in Parkinson’s disease
Source: Clin Neurophysiol. 2016 Apr;127(4):2010–9. doi: 10.1016/j.clinph.2016.01.015 (PMC4803022; doi:10.1016/j.clinph.2016.01.015)
Supplement: Supplementary Table S1 — Patient characteristics. [file mmc1.docx]

**Table S1.** Patient characteristics.

| **Case** | **Age (years) / gender** | **Disease duration (years)** | **Predominant symptoms  (in addition to akinesia)** | **UPDRS**  **left hemibody**  **ON/OFF medication** | **UPDRS right hemibody ON/OFF medication** | **Preoperative medication  (total daily dose)** | **Remarks** |
| --- | --- | --- | --- | --- | --- | --- | --- |
| 1 | 63/female | 9 | Tremor, pain | -/23 | -/9 | 4 mg rotigotine  1 mg rasagiline | OFF recording only |
| 2 | 58/male | 13 | Gait freezing | 6/10 | 4/7 | 1200 mg levodopa  400 mg amantadine  600 mg entacapone  1 mg rasagiline |  |
| 3 | 57/male | 17 | Gait impairment, pain, dyskinesias | 4/12 | 2/14 | 1550 mg levodopa  1600 mg entacapone  10 mg selegiline  200 mg amantadine |  |
| 4 | 60/male | 15 | Dyskinesias, gait freezing, tremor | 3/16 | 1/13 | 1300 mg levodopa  18 mg ropinirole  10 mg selegiline  2 mg amantadine |  |
| 5 | 61/male | 9 | Gait freezing, tremor | 2/8 | 1/6 | 1800 mg levodopa  500 µg pramipexole |  |
| 6 | 48/male | 11 | Gait freezing, tremor | 6/23 | 4/17 | 1250 mg levodopa  1 mg rasagiline  500 mg entacapone |  |
| 7 | 52/male | 12 | Dystonia | 2/8 | 1/7 | 950 mg levodopa  4 mg rotigotine  1 mg rasagiline |  |
| 8 | 51/male | 9 | Gait impairment, tremor | 7/16 | 6/13 | 800 mg levodopa  1 mg rasagiline |  |
| 9 | 59/female | 13 | Gait freezing, diskinesias | 2/- | 2/- | 500 mg levodopa  1 mg cabergoline  100 mg amantadine  2 mg apomorphine | ON recording only |
| 10 | 58/female | 14 | Gait freezing, pain, dyskinesias, motor fluctuations | 5/19 | 5/19 | 250 mg levodopa  4 mg pramipexole |  |
| 11 | 58/female | 10 | Dystonia, dyskinesia, motor fluctuations | 6/12 | 5/18 | 450 mg levodopa  3 mg pramipexole  2 mg rasagiline |  |
| 12 | 55/male | 15 | Tremor, freezing, motor fluctuations | 4/5 | 0/5 | 1000 mg levodopa  16 mg ropinirole  10 mg selegiline  100 mg amantadine |  |
| 13 | 64/female | 8 | Tremor, dyskinesias | 5/- | 6/- | 500 mg levodopa  17 mg ropinirole  300 mg amantadine  1 mg rasagline | ON recording only |
| 14 | 66/male | 8 | Freezing, motor fluctuations, dyskinesias, tremor | 4/- | 6/- | 800 mg levodopa  2 mg pramipexole  1 mg rasagline  200 mg amantadine  4-8 mg apomorphine | ON recording only |
| 15 | 40/male | 10 | Gait impairment, tremor | 2/7 | 1/7 | 1200 mg levodopa  5 mg pramipexole |  |
| 16 | 54/male | 8 | Gait impairment, dyskinesias | 3/6 | 3/15 | 1200 mg levodopa  4 mg cabergoline  800 mg entacapone  300 mg amantadine |  |
| 17 | 54/female | 10 | Freezing, tremor | 2/7 | 2/2 | 600 mg levodopa  2.6 mg pramipexole  100 mg amantadine |  |
| 18 | 61/female | 7 | Tremor, gait disturbance | 1/5 | 0/6 | 950 mg levodopa |  |
| 19 | 54/male | 15 |  | 5/13 | 5/16 | 1000 mg levodopa  1.5 mg pramipexole |  |
| 20 | 55/male | 11 | Tremor | 3/9 | 2/4 | 1000 mg entacapone  1625 mg levodopa  10 mg selegiline | ON recording only |
| 21 | 54/male | 6 | Gait disturbances, dyskinesia, tremor | 1/10 | 2/4 | 0.75 mg pramipexole  500 mg levodopa |  |
| 22 | 66/female | 11 | Freezing, dyskinesia, tremor | 6/15 | 3/10 | 562.5 mg levodopa  1200 mg entacapone  20 mg ropinirole | OFF recording only |
| 23 | 64/female | 15 | Dystonia | 3/7 | 2/7 | 187.5 mg levodopa  1.25 mg selegiline  16 mg rotigotine  200 mg amantadine  12 mg apomorphine |  |
| 24 | 54/male | 15 | Tremor, dyskinesia, freezing | 8/10 | 5/8 | 1200 mg stalevo  400 mg amantadine |  |
| 25 | 63/female | 11 |  | 4/14 | 3/11 | 400 mg levodopa  1 mg rasagiline  84 mg apomorphine | ON recording only |
| 26 | 63/male | 10 | Tremor, dyskinesia | 4/11 | 0/6 | 16 mg ropinirole  1 mg rasagiline  2 mg pergolide  250 mg levodopa | OFF recording only |
| 27 | 65/male | 16 | Dyskinesia | 4/10 | 0/7 | 462.5mg levodopa  100 mg amantadine |  |
| 28 | 70/male | 5 | Tremor | -/7 | -/11 | 500 mg levodopa  16 mg ropinirole | OFF left recording only |
| 29 | 58/male | 12 | Freezing, dyskinesia | 5/10 | 4/13 | 1500 mg levodopa | OFF recording only |
| 30 | 68/male | 12 | Freezing, dyskinesia, speech | 7/14 | 5/8 | 687.5 mg levodopa  1 mg rasagiline  24 mg ropinirole  4-24 mg apomorphine | OFF recording only |
| 31 | 60/male | 12 | Tremor | 5/8 | 1/7 | 32 mg ropinirole  937.5 mg levodopa  1 mg rasagiline  100 mg amantadine | OFF recording only |
| 32 | 59/male | 13 | Tremor, freezing | 3/10 | 3/8 | 2125 mg levodopa  400 mg tolcapone | OFF recording only |
| 33 | 54/male | 25 | Dystonia | 0/15 | 1/9 | 1600 mg levodopa  0.375 mg pramipexole  800 mg tolcapone | OFF recording only |

Hemibody UPDRS scores reflect the sum of scores for unilateral bradykinesia and rigidity items.
